# Voxel-based Specific Regional Analysis System for Alzheimer’s Disease (VSRAD) on 3-tesla Normal Database: Diagnostic Accuracy in Two Independent Cohorts with Early Alzheimer’s Disease

**DOI:** 10.14336/AD.2017.0818

**Published:** 2018-08-01

**Authors:** Daichi Sone, Etsuko Imabayashi, Norihide Maikusa, Masayo Ogawa, Noriko Sato, Hiroshi Matsuda, Japanese-Alzheimer’s Disease Neuroimaging Initiative

**Affiliations:** ^1^Department of Psychiatry, National Center of Neurology and Psychiatry, Tokyo, Japan; ^2^Integrative Brain Imaging Center, National Center of Neurology and Psychiatry, Tokyo, Japan; ^3^Department of Radiology, National Center of Neurology and Psychiatry, Tokyo, Japan

**Keywords:** Alzheimer’s disease, VSRAD, voxel-based morphometry, 3-tesla MRI

## Abstract

Voxel-based specific regional analysis system for Alzheimer’s disease (VSRAD) software is widely used in clinical practice in Alzheimer’s disease (AD). The existing VSRAD is based on the normal database with 1.5-tesla MRI scans (VSRAD-1.5T), and its utility for patients have undergone 3-tesla MRI is still controversial. We recruited 19 patients with early AD and 28 healthy controls who had undergone 3-tesla MRI scans at our institute (Cohort 1). We also used the 3-tesla MRI data of 30 patients with early AD and 13 healthy controls from the Japanese Alzheimer’s Disease Neuroimaging Initiative (Cohort 2). We also created a new VSRAD based on 65 normal subjects’ 3-tesla MRI scans (VSRAD-3T), and compared the detectability of AD between VSRAD-1.5T and VSRAD-3T, using receiver operating characteristic curve and area under the curve (AUC) analyses. As a result, there were no significant differences in the detectability of AD between VSRAD-3T and VSRAD-1.5T, except for the whole white matter atrophy score, which showed significantly better AUC in VSRAD-3T than in VSRAD-1.5T in both Cohort 1 (p=0.04) and 2 (p<0.01). Generally, there were better diagnostic values in Cohort 2 than in Cohort 1. The optimal cutoff values varied but were generally lower than in the previously published data. In conclusion, for patients with 3-tesla MRI, the detectability of early AD by the existing VSRAD was not different from that by the new VSRAD based on 3-tesla database. We should exercise caution when using the existing VSRAD for 3-tesla white matter analyses or for setting cutoff values.

Short Communication

Alzheimer’s disease (AD) is a common neuro-degenerative dementia, and hippocampal atrophy is a key morphological change that is useful for the diagnosis of AD in clinical practice [1]. The voxel-based specific regional analysis system for Alzheimer’s disease (VSRAD) is a diagnosis-aiding program, which runs on Windows, for voxel-based morphometry based on statistical parametric mapping (SPM8) and diffeomorphic anatomical registration using the exponentiated lie (DARTEL) [2]. VSRAD is widely used in current clinical practice in the treatment of AD [3]. Although 3-tesla MRI machines have become more and more common recently, the existing VSRAD is based on a normal database obtained from 1.5-tesla MRI scans [2]. A previous paper has raised the potential concern that the difference in magnetic field intensity can provide misdiagnostic information [4]. On the other hand, one study reported insignificant differences in hippocampal volumetry between 1.5- and 3- tesla MRI scans [5]. Thus, the utility of the existing VSRAD software for patients with 3-tesla MRI scans is still controversial, and should be accurately established to enable better clinical practice in the treatment of AD. In the current study, we demonstrated the diagnostic accuracy of the existing VSRAD using two independent cohorts with different 3-tesla MRI scans. In addition, we created new VSRAD software using a 3-tesla normal database from our institute, and compared the diagnostic values with those of the existing VSRAD.

## MATERIALS AND METHODS

### Patients Cohort 1 - recruitment at our institute

We recruited 19 patients with early AD and 28 healthy subjects at our institute. In this cohort, AD was diagnosed based on the clinical criteria for probable AD [11] and on the presence of an abnormal cortical accumulation of amyloid revealed by the visual assessment of ^11^C-PIB PET. We also visually confirmed that there was no abnormal accumulation of amyloid in healthy subjects. Table 1 describes their clinical demographics.

The MRI for this cohort was performed on a 3.0-tesla MR system (Verio, Siemens, Erlangen, Germany), and 3D sagittal T1-weighted magnetization prepared rapid acquisition with gradient echo (MPRAGE) images were obtained.

All subjects gave written consent to participate in the study, which was approved by the Institutional Review Board at the National Center of Neurology and Psychiatry.

### Patients Cohort 2 - J-ADNI data

In this cohort, we used data from the Japanese Alzheimer’s Disease Neuroimaging Initiative (J-ADNI). The clinical and imaging protocol is described elsewhere [6]. Most participants in the J-ADNI underwent 1.5-tesla MRI scans; we selected only participants who underwent 3-tesla MRI scans. The clinical demographics are also shown in Table 1.

**Table 1:** The demographics of Cohorts 1 and 2 and the normal databases for this study.

	Cohort 1 - Our Institute		Cohort 2 - J-ADNI		Normal Databases	

	Early AD (N=19)	Controls (N=28)	Early AD (N=30)	Controls (N=13)	VSRAD-3T (N=65)	VSRAD-1.5T ^*^ (N=80)
Age (mean ± SD)	69.8 ± 8.6	66.9 ± 7.9	74.2 ± 6.8	68.2 ± 6.0	70.3 ± 8.6	70.4 ± 7.8
Age (range)	53-81	54-86	61-83	61-80	54-85	54-86
Gender (M:F)	6:13	15:13	12:18	6:7	30:35	37:43
Global CDR (range)	0.5-1.0	0	0.5-1.0	0	N/A	N/A
MMSE (mean ± SD)	21.9 ± 4.5	29.3 ± 1.0	24.8 ± 2.5	29.8 ± 0.6	N/A	29.1 ± 1.2

* (Matsuda et al. AJNR Am J Neuroradiol. 2012)

### 3-tesla normal database for new VSRAD

We created new VSRAD software (VSRAD-3T) using the 3-tesla MRI scans of 65 healthy subjects without any neuropsychiatric disorders at our institute. There were no overlapping participants with either Cohort 1 or Cohort 2. In addition, their 3-tesla MRI scans were performed on different machines (Philips Medical Systems Achieva, Best, the Netherlands; and Verio, Siemens, Erlangen, Germany). A comparison of the demographics between both normal databases in VSRAD-3T and the existing VSRAD (VSRAD-1.5T) database is presented in Table 1.

### Image processing and score calculation

The 3D-T1 images of both Cohorts 1 and 2 were applied to the two different VSRAD programs (i.e., VSRAD-1.5T and VSRAD-3T). The image processing including SPM8 and DARTEL is the same in both VSRADs and was described elsewhere [2]. VSRAD generates the following scores: (1) a z-score of gray matter (GM) atrophy severity in the volume of interest (VOI) of AD (“Severity”); (2) the extent of GM atrophy in the VOI of AD (“Extent”); (3) the ratio of the extent of GM atrophy in the VOI to the whole brain (“Ratio”); (4) the maximum z-score of the severity of GM atrophy in the VOI of AD (“Maximum”); (5) the extent of GM atrophy in the whole brain (“Whole GM”); (6) the extent of atrophy in the white matter (WM) of the whole brain (“Whole WM”). A detailed explanation is also given in the same paper [2].

### Statistical Analyses

Statistical calculation was performed for each cohort separately. We evaluated the diagnostic accuracy of each score generated from both VSRADs using receiver operating characteristic curve and area under the curve (AUC) comparisons [7]. We compared the AUC values from both VSRADs using MedCalc Software ver. 17.4 (https://www.medcalc.org/).

In addition, the MedCalc Software calculated provisional optimal cut-off values and the sensitivity/specificity for each VSRAD score. However, since the optimal cutoff values are originally dependent on the prior probability of disease [8], clinicians have to use VSRAD in view of their patients’ prior probability of AD.

### Morphological comparison between the normal databases of both VSRADs

As supplementary analyses, we have performed voxel-based comparisons between the normal databases of both VSRADs. Both GM and WM images of the normal subjects, which had undergone same normalization (i.e. SPM8 and DARTEL), were compared by two-sample t-test model in SPM8 software. Differences meeting the following criteria were deemed significant: a height threshold of p<0.05 (familywise error) and an extent threshold of p<0.001 (false discovery rate).

**Table 2:** AUC values for differentiation of early AD from healthy controls using both VSRADs of ROC analysis

	Cohort 1 - Our Institute			Cohort 2 - J-ADNI		
	VSRAD-3T	VSRAD-1.5T	p-value	VSRAD-3T	VSRAD-1.5T	p-value
Severity	0.818	0.813	0.76	0.933	0.931	0.76
Extent	0.855	0.820	0.24	0.918	0.933	0.35
Ratio	0.847	0.806	0.22	0.903	0.918	0.30
Maximum	0.814	0.792	0.44	0.928	0.938	0.45
Whole GM	0.745	0.772	0.63	0.862	0.797	0.12
Whole WM	0.723	0.564	0.04*	0.865	0.662	<0.01*

AUC: area under the curve, ROC: receiver operating characteristic, GM: gray matter, WM: white matter.

## RESULTS

**Figure 1.**
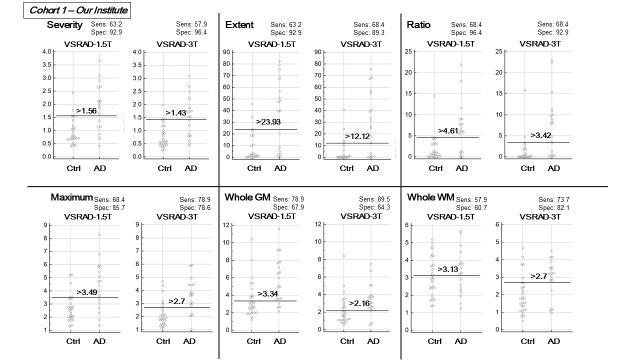
The beeswarm plots and diagnostic values at the optimal cutoff for each score on both VSRADs in Cohort 1.

**Figure 2.**
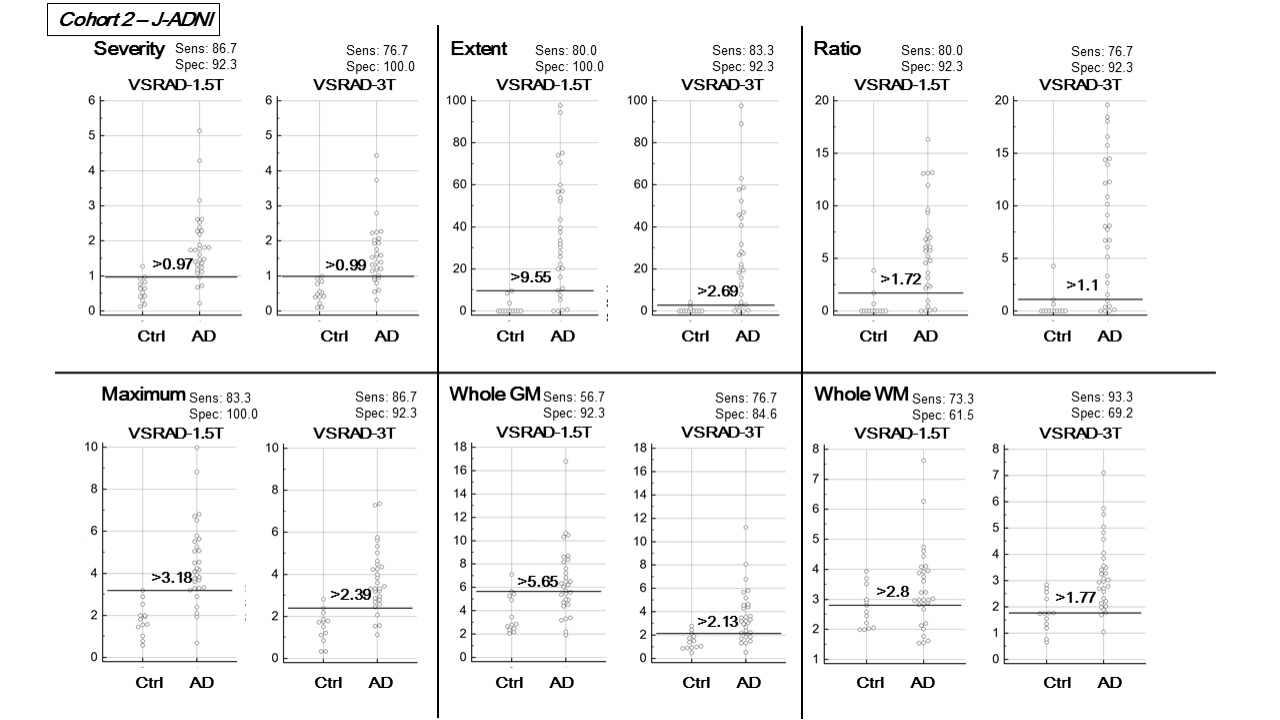
The beeswarm plots and diagnostic values at the optimal cutoff for each score on both VSRADs in Cohort 2.

According to the AUC comparison (Table 2), there were no significant differences in the detectability of AD between VSRAD-3T and -1.5T, except for the Whole WM score, which showed a significantly better AUC in VSRAD-3T than in VSRAD-1.5T in both Cohort 1 (p=0.04) and Cohort 2 (p<0.01). The beeswarm plots and diagnostic values at the optimal cutoff are shown in Figure 1 and Figure 2. Generally, there were better diagnostic values and better AUCs in Cohort 2 than in Cohort 1.

For clinical use of VSRAD, we have provided the detailed Supplementary Data including all the diagnostic values at every cutoff criterion, which contains the combined Cohort 1 and 2 data as well as each separated data (Table S1-3).

Furthermore, we found significant GM and WM differences between 80 normal subjects with 1.5-tesla scans and 65 with 3-tesla scans (Fig. S1). The 1.5-tesla group showed significant GM increase in the bilateral medial frontal lobes, GM decrease in the bilateral thalami, and WM increase in the bilateral frontal lobes.

## DISCUSSION

In the current study, we investigated the detectability of early AD patients using the existing VSRAD and a new VSRAD. For that, we used two independent cohorts with 3-tesla MRI scans. To the best of our knowledge, this is the first study focusing on the difference in the magnetic field intensity for use in VSRAD. Consequently, there were no significant differences in most scores in both cohorts. Therefore, in typical clinical practice, there would be no need to use the new VSRAD based on the 3-tesla normal database. Our results may support the previous report that revealed insignificant differences in hippocampal volumetry between 1.5- and 3- tesla MRI scans [5]. Another previous study compared tensor-based morphometry in AD between 1.5- and 3- tesla MRI and reported no significant difference in the detectability [9], which would accord with the results in the current study.

On the other hand, in both Cohort 1 and 2, we found significantly better detectability of AD in the Whole WM score by VSRAD-3T than by VSRAD-1.5T. Although WM volume reduction would also exist in AD [10], it is unusual to use the Whole WM score on VSRAD for the diagnosis of AD in clinical practice. Therefore, this result would provide little guidance for clinical practice. However, there may exist some difference in WM volumetry between 1.5- and 3- tesla MRI, which could be important in the field of neuroradiology. Notably, we found significant WM increase in normal subjects with 1.5-tesla scans as well as other interesting GM differences. Given the use of rigorous statistics, we consider these differences derive from the difference in magnetic field intensity. Possibly, VSRAD-1.5T may estimate less whole WM volumes and calculate high Whole WM atrophy score in normal subjects, which could lead to less AUC values for differentiation.

Another important finding in this study is the difference in the levels of detectability between the two cohorts. We found better diagnostic values and AUCs in Cohort 2 than in Cohort 1. The original paper on VSRAD reported that the Severity score showed high sensitivity (86.4%) and specificity (97.5%) for the diagnosis of very mild AD [2]. In the present study, Cohort 2 showed comparable diagnostic values, whereas less adequate results were found in Cohort 1. We speculate that the main reason for this inter-cohort difference would be aging. In Cohort 2, the patients with early AD were older than the controls by about 6 years (Table 1). This is because we enrolled all of the participants matching the criteria from the J-ADNI database without any intention or bias. The higher age in the Cohort 2 AD patients may have enhanced the atrophy scores in both VSRADs, since the hippocampal volume shrinks with aging [11], and there is overlapping atrophy in the hippocampal body and entorhinal cortex due to both AD and normal aging [12]. Thus, the effect of aging must be kept in mind in order to use VSRAD accurately. Additionally, the differing detectability levels between the two cohorts may have been influenced by background factors such as differences in the MRI devices or diagnostic criteria.

Moreover, the cutoff values for the diagnosis of AD in the current study were generally lower than those in the original paper (e.g., around “2” in Severity) [2]. In particular, the optimal cutoff values for Severity were consistently around “1” in both cohorts. Therefore, there may exist a tendency toward lower atrophy scores in 3-tesla MR images. The use of the cutoff value of “2” for the Severity score has high specificity but could lead to lower sensitivity and could cause patients potentially having early AD to be overlooked in 3-tesla MRI.

There are several reports of useful VSRAD applications focusing on the diagnosis of depression [13], prion disease [14], or the differentiation of AD and depression [15]. Some of those studies used 3-tesla MRI scans, and more studies using 3-tesla MRI and VSRAD software may emerge in the future. Our results may provide the existing VSRAD with a certain validation regarding GM analyses in 3-tesla MRI, whereas caution should be exercised in analyses of WM or in the determination of cutoff values.

This study has several limitations. First, the sample sizes in each cohort were relatively small, which might lead to the somewhat dispersed results. But we also confirmed several consistent results using the two independent cohorts. In addition, our new normal database lacked cognitive assessment (e.g., MMSE score), whereas the original VSRAD obtained such data [2]. However, we recruited only healthy subjects who reported no cognitive complaints for the normal database.

In conclusion, for patients who have undergone 3-tesla MRI, the detectability of early AD using the existing VSRAD is not different from that using the new VSRAD based on a 3-tesla normal database. Caution should be exercised when using the existing VSRAD for 3-tesla WM analyses or for the setting of cutoff values.

## SUPPLEMENTARY DATA


