# Supplementary material for: Voxel-based Specific Regional Analysis System for Alzheimer’s Disease (VSRAD) on 3-tesla Normal Database: Diagnostic Accuracy in Two Independent Cohorts with Early Alzheimer’s Disease
Source: Aging Dis. 2018 Aug 1;9(4):755–60. doi: 10.14336/AD.2017.0818 (PMC6065286; doi:10.14336/AD.2017.0818)
Supplement: Supplementary file 1 [file AD-9-4-755-s1.pdf]

SUPPLEMENTARY DATA

Short Communication

Voxel-based Specific Regional Analysis System for Alzheimer's Disease (VSRAD) on 3-tesla Normal Database: Diagnostic Accuracy in Two Independent Cohorts with Early Alzheimer's Disease

Daichi Sone[1,2], Etsuko Imabayashi[2], Norihide Maikusa[2], Masayo Ogawa[2], Noriko Sato[3], Hiroshi Matsuda[2,*], Japanese-Alzheimer's Disease Neuroimaging Initiative

[1]Department of Psychiatry, National Center of Neurology and Psychiatry, Tokyo, Japan
[2]Integrative Brain Imaging Center, National Center of Neurology and Psychiatry, Tokyo, Japan
[3]Department of Radiology, National Center of Neurology and Psychiatry, Tokyo, Japan

© 2017 Sone D. et al. Published online at http://dx.doi.org/10.14336/AD.2017.0818

SUPPLEMENTARY DATA

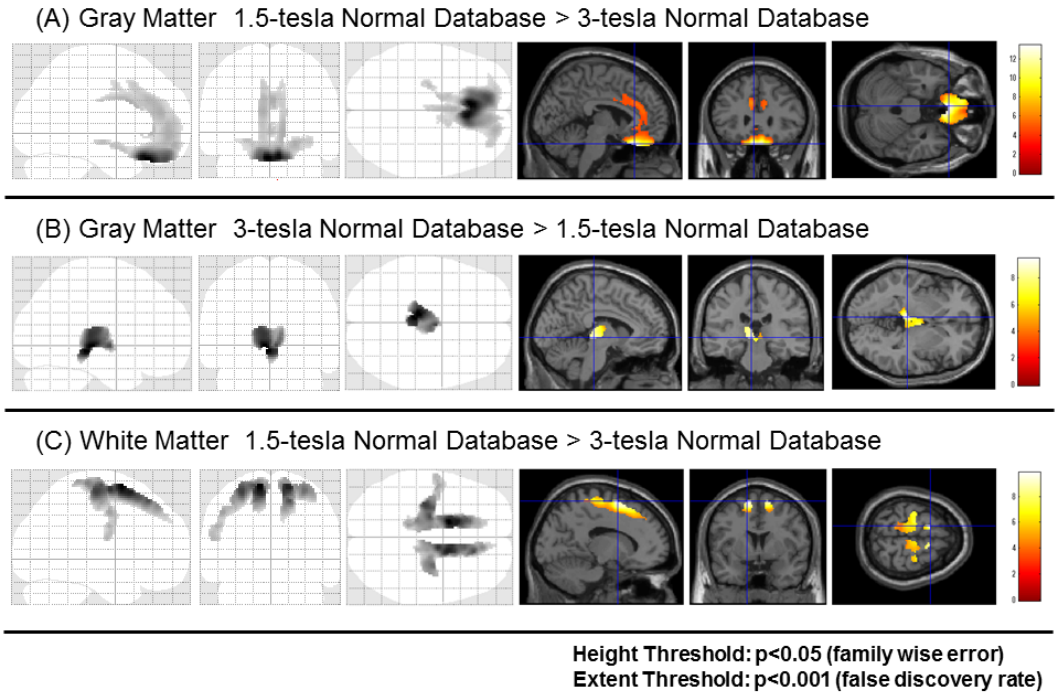

Supplemental Figure 1. Significant morphological differences between the normal databases of both VSRADs.

© 2017 Sone D. et al. Published online at http://dx.doi.org/10.14336/AD.2017.0818

SUPPLEMENTARY DATA

Table S1. Diagnostic values at every criterion, calculated from combined Cohort 1 and 2 data (49 with early AD and 41 healthy subjects). Yellow highlight denotes the provisional optimal cutoff value.

VSRAD-1.5T Severity			VSRAD-3T Severity		
**Criterion**	**Sensitivity**	**Specificity**	**Criterion**	**Sensitivity**	**Specificity**
≥0.13	100	0	≥0.1	100	0
>0.19	100	4.88	>0.28	100	12.2
>0.22	97.96	4.88	>0.31	97.96	12.2
>0.39	97.96	9.76	>0.42	97.96	24.39
>0.41	95.92	12.2	>0.43	95.92	26.83
>0.67	95.92	41.46	>0.49	95.92	41.46
>0.68	91.84	41.46	>0.51	93.88	41.46
>0.7	91.84	48.78	>0.53	93.88	46.34
>0.72	89.8	51.22	>0.55	91.84	46.34
>0.73	87.76	51.22	>0.58	91.84	53.66
>0.84	87.76	65.85	>0.59	89.8	56.1
>0.86	85.71	65.85	>0.61	89.8	60.98
>0.92	85.71	68.29	>0.62	87.76	60.98
>0.96	83.67	70.73	>0.69	87.76	63.41
>1.05	83.67	75.61	>0.78	85.71	65.85
>1.09	81.63	78.05	>0.8	81.63	68.29
>1.11	81.63	80.49	>0.87	81.63	73.17
>1.15	75.51	80.49	>0.88	79.59	73.17
>1.18	73.47	82.93	>0.9	77.55	75.61
>1.24	71.43	82.93	>0.93	77.55	80.49
>1.27	71.43	85.37	>0.98	73.47	80.49
>1.36	67.35	85.37	>0.99	73.47	82.93
>1.38	65.31	87.8	>1.01	71.43	85.37
>1.48	59.18	87.8	>1.14	69.39	85.37
>1.56	59.18	95.12	>1.17	67.35	87.8
>1.74	51.02	95.12	>1.19	67.35	90.24
>1.77	48.98	97.56	>1.32	59.18	90.24
>2.39	22.45	97.56	>1.33	59.18	92.68
>2.43	22.45	100	>1.4	53.06	92.68
>5.14	0	100	>1.43	53.06	97.56
			>1.94	26.53	97.56
			>1.99	26.53	100
			>4.44	0	100

VSRAD-1.5T Extent			VSRAD-3T Extent		
**Criterion**	**Sensitivity**	**Specificity**	**Criterion**	**Sensitivity**	**Specificity**
≥0	100	0	≥0	100	0
>0	91.84	41.46	>0	91.84	60.98
>0.12	91.84	48.78	>0.06	91.84	65.85

© 2017 Sone D. et al. Published online at http://dx.doi.org/10.14336/AD.2017.0818

SUPPLEMENTARY DATA

>0.31	89.8	48.78	>0.24	85.71	65.85
>0.43	89.8	53.66	>0.55	83.67	68.29
>0.61	87.76	53.66	>1.29	83.67	78.05
>1.29	87.76	60.98	>2.2	79.59	78.05
>1.65	85.71	60.98	>2.69	79.59	82.93
>2.14	85.71	68.29	>4.1	71.43	82.93
>2.57	83.67	68.29	>6.06	71.43	87.8
>3.61	83.67	75.61	>7.83	69.39	87.8
>8.26	75.51	75.61	>9.61	69.39	90.24
>9.55	75.51	80.49	>11.38	67.35	90.24
>9.73	73.47	80.49	>12.12	67.35	92.68
>10.65	73.47	85.37	>12.61	63.27	92.68
>16.16	69.39	85.37	>14.08	63.27	95.12
>18.67	69.39	90.24	>22.09	51.02	95.12
>22.22	61.22	90.24	>24.3	51.02	97.56
>23.93	61.22	95.12	>40.7	28.57	97.56
>33.48	48.98	95.12	>40.76	28.57	100
>34.39	48.98	97.56	>97.61	0	100
>43.45	38.78	97.56			
>45.72	38.78	100			
>97.67	0	100			

**VSRAD-1.5T**			**VSRAD-3T**		
**Ratio**			**Ratio**		
**Criterion**	**Sensitivity**	**Specificity**	**Criterion**	**Sensitivity**	**Specificity**
≥0	100	0	≥0	100	0
>0	91.84	41.46	>0	91.84	60.98
>0.05	91.84	48.78	>0.03	91.84	65.85
>0.12	87.76	48.78	>0.21	85.71	65.85
>0.32	87.76	56.1	>0.22	85.71	68.29
>0.46	83.67	56.1	>0.37	79.59	68.29
>0.85	83.67	70.73	>0.41	79.59	70.73
>1.1	77.55	70.73	>0.47	77.55	70.73
>1.31	77.55	75.61	>0.77	77.55	78.05
>1.38	75.51	75.61	>0.99	75.51	78.05
>1.72	75.51	78.05	>1.1	75.51	80.49
>2.46	69.39	78.05	>1.55	71.43	80.49
>3.1	69.39	85.37	>2.22	71.43	87.8
>3.14	67.35	85.37	>2.65	69.39	87.8
>3.31	67.35	87.8	>2.89	69.39	90.24
>3.63	65.31	87.8	>3.32	67.35	90.24
>4.3	65.31	95.12	>3.42	67.35	92.68
>4.57	63.27	95.12	>3.43	65.31	92.68
>4.61	63.27	97.56	>4.73	65.31	97.56
>13.16	6.12	97.56	>15.75	12.24	97.56
>14.4	6.12	100	>15.87	12.24	100
>21.91	0	100	>22.92	0	100

© 2017 Sone D. et al. Published online at http://dx.doi.org/10.14336/AD.2017.0818

SUPPLEMENTARY DATA

VSRAD-1.5T Maximum Criterion	Sensitivity	Specificity
≥0.58	100	0
>0.58	100	2.44
>0.68	97.96	2.44
>1.3	97.96	9.76
>1.37	95.92	9.76
>1.79	95.92	26.83
>1.81	93.88	29.27
>1.93	93.88	34.15
>1.94	91.84	34.15
>2.08	91.84	48.78
>2.1	89.8	48.78
>2.27	89.8	53.66
>2.39	85.71	53.66
>2.65	85.71	65.85
>2.68	83.67	65.85
>2.89	83.67	78.05
>2.9	81.63	78.05
>3.02	81.63	80.49
>3.06	79.59	80.49
>3.18	79.59	82.93
>3.33	69.39	82.93
>3.49	69.39	90.24
>3.55	67.35	92.68
>4.55	40.82	92.68
>4.6	40.82	95.12
>5.1	30.61	95.12
>5.23	30.61	100
>9.98	0	100

VSRAD-3T Maximum Criterion	Sensitivity	Specificity
≥0.34	100	0
>0.85	100	7.32
>1.03	97.96	7.32
>1.12	97.96	12.2
>1.13	95.92	12.2
>1.51	95.92	31.71
>1.57	91.84	31.71
>1.92	91.84	60.98
>2.05	89.8	60.98
>2.06	89.8	63.41
>2.08	87.76	63.41
>2.12	87.76	65.85
>2.13	85.71	65.85
>2.19	85.71	73.17
>2.2	83.67	73.17
>2.4	83.67	78.05
>2.46	81.63	78.05
>2.51	81.63	80.49
>2.57	79.59	80.49
>2.7	77.55	82.93
>2.79	75.51	82.93
>2.82	75.51	85.37
>2.87	71.43	85.37
>2.91	71.43	87.8
>3.02	65.31	87.8
>3.08	65.31	90.24
>3.14	63.27	90.24
>3.18	63.27	92.68
>3.89	36.73	92.68
>3.95	36.73	95.12
>4.36	28.57	95.12
>4.38	28.57	97.56
>4.64	18.37	97.56
>4.69	18.37	100
>7.36	0	100

VSRAD-1.5T Whole GM Criterion	Sensitivity	Specificity
≥1.22	100	0
>1.91	100	9.76
>1.92	97.96	9.76
>2.17	97.96	24.39

VSRAD-3T Whole GM Criterion	Sensitivity	Specificity
≥0.5	100	0
>0.5	100	2.44
>0.53	97.96	4.88
>0.73	93.88	4.88

© 2017 Sone D. et al. Published online at http://dx.doi.org/10.14336/AD.2017.0818

SUPPLEMENTARY DATA

>2.19	93.88	24.39	>1.23	93.88	41.46
>2.62	93.88	39.02	>1.32	89.8	41.46
>2.66	89.8	39.02	>1.41	89.8	46.34
>2.86	89.8	53.66	>1.49	87.76	46.34
>3.07	87.76	56.1	>1.6	87.76	53.66
>3.17	87.76	60.98	>1.69	85.71	53.66
>3.32	83.67	60.98	>1.84	85.71	60.98
>3.34	83.67	63.41	>1.87	81.63	60.98
>3.39	81.63	63.41	>2.13	81.63	68.29
>3.47	81.63	65.85	>2.16	79.59	70.73
>3.66	75.51	65.85	>2.38	71.43	70.73
>4	75.51	73.17	>2.44	71.43	73.17
>4.73	69.39	73.17	>2.47	69.39	75.61
>4.74	69.39	75.61	>2.56	65.31	75.61
>4.91	67.35	75.61	>2.57	65.31	78.05
>4.92	67.35	78.05	>2.68	61.22	78.05
>5.09	59.18	78.05	>2.76	61.22	82.93
>5.36	59.18	82.93	>2.95	59.18	82.93
>5.38	57.14	85.37	>2.97	59.18	85.37
>5.42	57.14	87.8	>3.06	53.06	85.37
>5.58	53.06	87.8	>3.12	53.06	87.8
>5.65	53.06	90.24	>3.33	44.9	87.8
>5.99	48.98	90.24	>3.43	44.9	90.24
>6.03	48.98	92.68	>3.48	42.86	90.24
>6.84	32.65	92.68	>3.54	42.86	92.68
>7.12	32.65	95.12	>4.32	26.53	92.68
>7.82	22.45	95.12	>4.33	26.53	95.12
>8.05	22.45	97.56	>4.65	20.41	95.12
>10.31	8.16	97.56	>5	20.41	97.56
>10.4	8.16	100	>8.07	2.04	97.56
>16.8	0	100	>8.4	2.04	100
			>11.21	0	100

**VSRAD-1.5T Whole WM**			**VSRAD-3T Whole WM**		
**Criterion**	**Sensitivity**	**Specificity**	**Criterion**	**Sensitivity**	**Specificity**
≥1.24	100	0	≥0.51	100	0
>1.24	97.96	0	>0.94	100	14.63
>1.41	97.96	4.88	>1.05	95.92	14.63
>1.62	89.8	4.88	>1.08	95.92	17.07
>1.76	89.8	12.2	>1.09	93.88	19.51
>1.77	87.76	12.2	>1.14	93.88	21.95
>1.88	87.76	14.63	>1.15	91.84	21.95
>1.96	85.71	14.63	>1.2	91.84	24.39
>1.99	85.71	19.51	>1.21	89.8	24.39
>2.01	83.67	19.51	>1.56	89.8	41.46
>2.06	83.67	26.83	>1.7	87.76	41.46

© 2017 Sone D. et al. Published online at http://dx.doi.org/10.14336/AD.2017.0818

SUPPLEMENTARY DATA

>2.19	77.55	26.83	>1.77	87.76	53.66
>2.22	77.55	29.27	>1.78	85.71	58.54
>2.25	75.51	29.27	>1.86	85.71	60.98
>2.39	75.51	31.71	>2.03	79.59	60.98
>2.4	73.47	31.71	>2.05	79.59	63.41
>2.58	73.47	46.34	>2.13	77.55	63.41
>2.67	69.39	46.34	>2.14	77.55	65.85
>2.8	69.39	51.22	>2.22	75.51	65.85
>2.84	65.31	51.22	>2.25	75.51	68.29
>2.89	63.27	53.66	>2.26	73.47	68.29
>2.91	61.22	56.1	>2.3	73.47	70.73
>2.96	59.18	56.1	>2.33	71.43	70.73
>2.97	59.18	58.54	>2.56	71.43	78.05
>2.98	57.14	60.98	>2.59	69.39	78.05
>2.99	53.06	60.98	>2.69	69.39	80.49
>3	53.06	63.41	>2.7	67.35	82.93
>3.02	51.02	63.41	>2.71	65.31	85.37
>3.13	51.02	65.85	>2.82	59.18	85.37
>3.23	46.94	65.85	>2.84	59.18	87.8
>3.24	44.9	68.29	>2.94	55.1	87.8
>3.28	42.86	68.29	>2.95	55.1	90.24
>3.52	42.86	73.17	>3.56	24.49	90.24
>3.61	38.78	73.17	>3.74	24.49	95.12
>3.74	38.78	80.49	>4.14	14.29	95.12
>3.92	24.49	80.49	>4.15	14.29	97.56
>3.93	24.49	82.93	>4.57	10.2	97.56
>4.1	18.37	82.93	>4.69	10.2	100
>4.33	18.37	90.24	>7.11	0	100
>4.61	12.24	90.24			
>4.67	12.24	92.68			
>4.73	10.2	92.68			
>4.75	10.2	95.12			
>4.84	8.16	95.12			
>5.19	8.16	100			
>7.63	0	100			

© 2017 Sone D. et al. Published online at http://dx.doi.org/10.14336/AD.2017.0818

SUPPLEMENTARY DATA

Table S2. Diagnostic values at every cr
iterion, calculated from only Cohort 1 data
(19 with early AD and 28 healthy subjects). Yellow highlight denotes the provisional optimal cutoff value.

VSRAD-1.5T Severity			VSRAD-3T Severity		
**Criterion**	**Sensitivity**	**Specificity**	**Criterion**	**Sensitivity**	**Specificity**
≥0.39	100	0	≥0.25	100	0
>0.39	100	3.57	>0.4	100	14.29
>0.41	94.74	3.57	>0.43	94.74	17.86
>0.67	94.74	32.14	>0.49	94.74	35.71
>0.68	89.47	32.14	>0.51	89.47	35.71
>0.72	89.47	46.43	>0.61	89.47	57.14
>0.73	84.21	46.43	>0.62	84.21	57.14
>0.84	84.21	60.71	>0.69	84.21	60.71
>0.86	78.95	60.71	>0.78	78.95	60.71
>1.11	78.95	75	>0.8	73.68	64.29
>1.15	68.42	75	>0.87	73.68	67.86
>1.18	68.42	78.57	>0.88	68.42	67.86
>1.38	63.16	82.14	>1.19	68.42	85.71
>1.56	63.16	92.86	>1.21	63.16	85.71
>1.65	57.89	92.86	>1.33	63.16	89.29
>1.77	57.89	96.43	>1.4	57.89	89.29
>2.13	26.32	96.43	>1.43	57.89	96.43
>2.43	26.32	100	>1.88	26.32	96.43
>3.67	0	100	>1.99	26.32	100
			>3.09	0	100

VSRAD-1.5T Extent			VSRAD-3T Extent		
**Criterion**	**Sensitivity**	**Specificity**	**Criterion**	**Sensitivity**	**Specificity**
≥0	100	0	≥0	100	0
>0	89.47	25	>0	94.74	53.57
>1.29	89.47	53.57	>0.06	94.74	60.71
>1.65	84.21	53.57	>0.18	84.21	60.71
>2.14	84.21	64.29	>0.55	78.95	64.29
>2.57	78.95	64.29	>1.29	78.95	75
>3.43	78.95	71.43	>1.35	73.68	75
>8.26	68.42	71.43	>2.39	73.68	78.57
>18.67	68.42	85.71	>3.12	68.42	78.57
>20.44	63.16	85.71	>12.12	68.42	89.29
>23.93	63.16	92.86	>12.3	63.16	89.29
>29.13	57.89	92.86	>14.08	63.16	92.86
>34.39	57.89	96.43	>17.99	57.89	92.86
>39.9	47.37	96.43	>24.3	57.89	96.43
>45.72	47.37	100	>40.21	26.32	96.43

© 2017 Sone D. et al. Published online at http://dx.doi.org/10.14336/AD.2017.0818

SUPPLEMENTARY DATA

>82.37	0	100	>40.76	26.32	100
			>75.64	0	100

VSRAD-1.5T Ratio			VSRAD-3T Ratio		
**Criterion**	**Sensitivity**	**Specificity**	**Criterion**	**Sensitivity**	**Specificity**
≥0	100	0	≥0	100	0
>0	89.47	25	>0	94.74	53.57
>0.32	89.47	46.43	>0.03	94.74	60.71
>0.46	84.21	46.43	>0.21	84.21	60.71
>0.85	84.21	64.29	>0.22	84.21	64.29
>1.1	73.68	64.29	>0.35	73.68	64.29
>1.31	73.68	71.43	>0.77	73.68	75
>1.38	68.42	71.43	>1.24	68.42	75
>4.61	68.42	96.43	>3.42	68.42	92.86
>11.65	10.53	96.43	>3.43	63.16	92.86
>14.4	10.53	100	>4.73	63.16	96.43
>21.91	0	100	>15.42	10.53	96.43
			>15.87	10.53	100
			>22.92	0	100

VSRAD-1.5T Maximum			VSRAD-3T Maximum		
**Criterion**	**Sensitivity**	**Specificity**	**Criterion**	**Sensitivity**	**Specificity**
≥1.3	100	0	≥1.03	100	0
>1.3	100	3.57	>1.03	94.74	0
>1.37	94.74	3.57	>1.92	94.74	53.57
>1.79	94.74	17.86	>2.05	89.47	53.57
>1.81	89.47	17.86	>2.12	89.47	60.71
>2.27	89.47	42.86	>2.13	84.21	60.71
>2.32	84.21	42.86	>2.19	84.21	67.86
>2.65	84.21	57.14	>2.2	78.95	67.86
>2.68	78.95	57.14	>2.7	78.95	78.57
>3.02	78.95	75	>2.85	73.68	78.57
>3.33	68.42	75	>2.91	73.68	82.14
>3.49	68.42	85.71	>3.02	63.16	82.14
>3.55	63.16	89.29	>3.08	63.16	85.71
>4.42	47.37	89.29	>3.14	57.89	85.71
>4.6	47.37	92.86	>3.18	57.89	89.29
>5.1	31.58	92.86	>3.89	31.58	89.29
>5.23	31.58	100	>4.38	31.58	96.43
>8.25	0	100	>4.44	15.79	96.43
			>4.69	15.79	100
			>5.9	0	100

© 2017 Sone D. et al. Published online at http://dx.doi.org/10.14336/AD.2017.0818

SUPPLEMENTARY DATA

VSRAD-1.5T Whole GM			VSRAD-3T Whole GM		
**Criterion**	**Sensitivity**	**Specificity**	**Criterion**	**Sensitivity**	**Specificity**
≥1.22	100	0	≥0.53	100	0
>2.13	100	28.57	>0.53	100	3.57
>2.19	94.74	28.57	>0.73	89.47	3.57
>2.62	94.74	42.86	>2.16	89.47	64.29
>2.66	84.21	42.86	>2.2	84.21	64.29
>2.86	84.21	53.57	>2.47	78.95	67.86
>3.07	78.95	57.14	>2.56	68.42	67.86
>3.34	78.95	67.86	>2.57	68.42	71.43
>3.66	63.16	67.86	>2.68	63.16	71.43
>4.74	63.16	82.14	>2.97	63.16	78.57
>5.09	47.37	82.14	>3.04	52.63	78.57
>5.38	47.37	89.29	>3.12	52.63	82.14
>5.99	42.11	89.29	>3.33	47.37	82.14
>6.03	42.11	92.86	>3.54	47.37	89.29
>7.82	15.79	92.86	>3.95	21.05	89.29
>8.05	15.79	96.43	>5	21.05	96.43
>9.2	5.26	96.43	>7.47	0	96.43
>10.4	5.26	100	>8.4	0	100
>11.56	0	100			

VSRAD-1.5T Whole WM			VSRAD-3T Whole WM		
**Criterion**	**Sensitivity**	**Specificity**	**Criterion**	**Sensitivity**	**Specificity**
≥1.24	100	0	≥0.51	100	0
>1.24	94.74	0	>0.94	100	14.29
>1.41	94.74	7.14	>0.95	94.74	14.29
>1.54	89.47	7.14	>1.08	94.74	17.86
>1.88	89.47	21.43	>1.09	89.47	21.43
>1.96	84.21	21.43	>1.14	89.47	25
>2.02	84.21	25	>1.21	78.95	25
>2.25	73.68	25	>2.25	78.95	67.86
>2.39	73.68	28.57	>2.26	73.68	67.86
>2.4	68.42	28.57	>2.7	73.68	82.14
>2.49	68.42	42.86	>2.92	57.89	82.14
>2.6	63.16	42.86	>2.95	57.89	85.71
>2.89	63.16	50	>3.56	21.05	85.71
>2.91	57.89	50	>3.74	21.05	92.86
>3.13	57.89	60.71	>4.14	5.26	92.86
>3.22	52.63	60.71	>4.15	5.26	96.43
>3.24	52.63	64.29	>4.51	0	96.43
>3.28	47.37	64.29	>4.69	0	100
>3.47	47.37	67.86			
>3.55	42.11	67.86			

© 2017 Sone D. et al. Published online at http://dx.doi.org/10.14336/AD.2017.0818

SUPPLEMENTARY DATA

>3.74	42.11	75
>3.87	21.05	75
>4.33	21.05	85.71
>4.47	15.79	85.71
>4.75	15.79	92.86
>4.84	10.53	92.86
>5.19	10.53	100
>5.65	0	100

Table S3. Diagnostic values at every criterion, calculated from only Cohort 2 data (30 with early AD and 13 healthy subjects). Yellow highlight denotes the provisional optimal cutoff value.

**VSRAD-1.5T Severity**			**VSRAD-3T Severity**		
**Criterion**	**Sensitivity**	**Specificity**	**Criterion**	**Sensitivity**	**Specificity**
≥0.13	100	0	≥0.1	100	0
>0.19	100	15.38	>0.22	100	23.08
>0.22	96.67	15.38	>0.31	96.67	23.08
>0.64	96.67	61.54	>0.53	96.67	61.54
>0.72	90	61.54	>0.55	93.33	61.54
>0.92	90	84.62	>0.56	93.33	69.23
>0.96	86.67	84.62	>0.59	90	69.23
>0.97	86.67	92.31	>0.78	90	76.92
>1.24	73.33	92.31	>0.8	86.67	76.92
>1.27	73.33	100	>0.84	86.67	84.62
>5.14	0	100	>0.9	83.33	84.62
			>0.92	83.33	92.31
			>0.98	76.67	92.31
			>0.99	76.67	100
			>4.44	0	100

**VSRAD-1.5T Extent**			**VSRAD-3T Extent**		
**Criterion**	**Sensitivity**	**Specificity**	**Criterion**	**Sensitivity**	**Specificity**
≥0	100	0	≥0	100	0
>0	93.33	76.92	>0	90	76.92
>0.61	86.67	76.92	>0.24	86.67	76.92
>3.61	86.67	84.62	>1.22	86.67	84.62
>7.34	80	84.62	>2.2	83.33	84.62
>9.55	80	100	>2.69	83.33	92.31
>97.67	0	100	>4.1	73.33	92.31
			>4.16	73.33	100
			>97.61	0	100

**VSRAD-1.5T**	**VSRAD-3T**

© 2017 Sone D. et al. Published online at http://dx.doi.org/10.14336/AD.2017.0818

SUPPLEMENTARY DATA

Ratio			Ratio		
**Criterion**	**Sensitivity**	**Specificity**	**Criterion**	**Sensitivity**	**Specificity**
≥0	100	0	≥0	100	0
>0	93.33	76.92	>0	90	76.92
>0.44	83.33	76.92	>0.47	80	76.92
>0.67	83.33	84.62	>0.64	80	84.62
>0.93	80	84.62	>0.99	76.67	84.62
>1.72	80	92.31	>1.1	76.67	92.31
>3.63	63.33	92.31	>3.32	66.67	92.31
>3.85	63.33	100	>4.27	66.67	100
>16.3	0	100	>19.59	0	100

VSRAD-1.5T Whole GM			VSRAD-3T Whole GM		
**Criterion**	**Sensitivity**	**Specificity**	**Criterion**	**Sensitivity**	**Specificity**
≥1.92	100	0	≥0.5	100	0
>1.92	96.67	0	>0.5	100	7.69
>2.17	96.67	15.38	>0.53	96.67	7.69
>2.19	93.33	15.38	>1.05	96.67	46.15
>2.85	93.33	53.85	>1.32	90	46.15
>3.39	83.33	53.85	>1.41	90	53.85
>3.47	83.33	61.54	>1.49	86.67	53.85
>4.73	73.33	61.54	>1.51	86.67	61.54
>4.92	73.33	69.23	>1.69	83.33	61.54
>4.96	66.67	69.23	>1.72	83.33	69.23
>5.28	66.67	76.92	>1.87	76.67	69.23
>5.38	63.33	76.92	>2.13	76.67	84.62
>5.42	63.33	84.62	>2.38	63.33	84.62
>5.58	56.67	84.62	>2.44	63.33	92.31
>5.65	56.67	92.31	>2.67	60	92.31
>6.84	33.33	92.31	>2.76	60	100
>7.12	33.33	100	>11.21	0	100
>16.8	0	100			

VSRAD-1.5T Whole WM			VSRAD-3T Whole WM		
**Criterion**	**Sensitivity**	**Specificity**	**Criterion**	**Sensitivity**	**Specificity**
≥1.54	100	0	≥0.66	100	0
>1.77	86.67	0	>0.77	100	15.38
>1.99	86.67	15.38	>1.05	96.67	15.38
>2.01	83.33	15.38	>1.56	96.67	38.46
>2.06	83.33	30.77	>1.7	93.33	38.46
>2.19	76.67	30.77	>1.77	93.33	69.23
>2.58	76.67	53.85	>2.22	73.33	69.23
>2.67	73.33	53.85	>2.3	73.33	76.92
>2.8	73.33	61.54	>2.33	70	76.92

© 2017 Sone D. et al. Published online at http://dx.doi.org/10.14336/AD.2017.0818

SUPPLEMENTARY DATA

>2.89	63.33	61.54	>2.56	70	84.62
>2.91	63.33	69.23	>2.7	63.33	84.62
>2.99	50	69.23	>2.71	60	92.31
>3	50	76.92	>2.78	56.67	92.31
>3.24	40	76.92	>2.84	56.67	100
>3.52	40	84.62	>7.11	0	100
>3.61	36.67	84.62			
>3.7	36.67	92.31			
>3.92	26.67	92.31			
>3.93	26.67	100			
>7.63	0	100			

© 2017 Sone D. et al. Published online at http://dx.doi.org/10.14336/AD.2017.0818
